# Limited Utility of Chemotherapy for Pulmonary Langerhans Cell Histiocytosis Due to Unclear Diagnostic Criteria

**DOI:** 10.7759/cureus.22324

**Published:** 2022-02-17

**Authors:** Tanjot S Saini, Sharad K Dass, Simran Dhillon

**Affiliations:** 1 Internal Medicine, University of California San Francisco, Fresno, USA; 2 Internal Medicine, O'Connor Hospital, San Jose, USA

**Keywords:** hypereosinophilia syndrome, hypereosinophilia, cd-1a, asthma, plch, vemurafenib, cladribine, chemotherapy, langerhans cell histiocytosis(lch)

## Abstract

Pulmonary Langerhans cell histiocytosis (PLCH) is a rare interstitial lung disease that affects young adults and is typically misdiagnosed or presents concurrently with more common respiratory conditions such as asthma or chronic obstructive lung disease (COPD). A combination of imaging, tissue biopsy, and clinical presentation is used for diagnosis since no definitive criteria have been established. Current standards are based on vague descriptors such as clustering of histologic markers but lack quantitative analysis. In this case report, we present a patient who was initially diagnosed with PLCH, but the lack of unanimity for diagnostic requirements led to conflicting diagnoses between institutions which may have prevented optimal care for the patient. The disparity limited new, alternative therapies for our patient that may have been beneficial since he was clinically not improving with smoking cessation and standard of care for obstructive lung diseases. However, quantitative endorsements for tissue analysis, such as requiring more than 30 Cd-1a stained Langerhans cells per high power field (HPF), may reduce discrepancies associated with current techniques. It is imperative that clear standards are established due to the unique treatment these patients require that is atypical from other pulmonary diseases such as asthma and COPD. New chemotherapeutic regimens such as cladribine and vemurafenib require oncologic care and have more broad side effects than typical pulmonary treatments, which emphasize the need for accurate diagnoses before starting treatment. Existing standards have created circumstances where PLCH is a differential but cannot be ruled out due to unclear criteria and limited research.

## Introduction

Pulmonary Langerhans cell (LC) histiocytosis (PLCH) has an unknown cause and is a rare neoplastic disorder characterized by the infiltration of lungs and various organs by bone marrow-derived Langerhans cells with an accompanying strong inflammatory response. PLCH affects males and females equally in the United States, with a peak frequency of 20-40 years of age [1,2]. Caucasians are most commonly affected, but no correlations to occupation or geographic areas have been found. Current or previous tobacco-related smoking was prevalent in up to 97% of patients [3]. The rare prevalence of the disease was emphasized at the Denver Specialized Center of Research program in interstitial lung disease, where only 2% of patients presented with PLCH, but 5% were diagnosed via biopsy [4]. This suggests PLCH requires further studies with a large sample size to better understand its clinical characteristics and prevent misdiagnoses [5].

The pathogenesis of PLCH is unknown, but it is hypothesized to be due to an increase in IgG in bronchoalveolar lavage fluid or increases in immune complexes throughout the body [6]. The near direct correlation to cigarette smoking implies a causative role. Other hypotheses include a reactive polyclonal process induced by antigens and increased bombesin levels in smokers [7]. Bombesin is a neuroendocrine cell that stimulates cytokine stimulation and is chemotactic for monocytes, the origin of Langerhans cells [8].

PLCH patients experience common respiratory symptoms such as nonproductive cough, dyspnea, and pleuritic chest pain. More concerning symptoms include hemoptysis, diabetes insipidus, benign tumors, and cystic bone lesions commonly involving flat bones [9]. Pleuritic chest pain can, in some cases, be attributed to cystic bone lesions involving the ribs [10]. Major complications include spontaneous pneumothorax, pulmonary arteriopathy, and pulmonary hypertension. The degree of pulmonary hypertension has the most significant impact on the longevity of PLCH patients [11].

Langerhans cells differentiate from the monocyte-macrophage line and are identified via immunostaining or electron microscopy [4]. Immunostaining is routinely favored over electron microscopy due to the lower cost and the low availability of specific immunostains in electron microscopy. This limits the practical utilization of Birbeck granules which are cytoplasmic organelles unique to Langerhan cells [12]. Immunohistochemical staining for langerin, S100, and a presence of Cd-1a on the cell surface are unique to Langerhans cells that play an essential part in immunostaining.

The initial diagnostic tool is a high-resolution computed tomography (CT), which will show cysts and nodules predominantly in the middle and upper lung zones [13]. Tissue immunostaining using the most specific marker, Cd-1a, is used as an adjunct in difficult cases to differentiate Langerhans cells from other histiocytes [14]. Bronchoalveolar lavage (BAL) is uncommonly used to diagnose PLCH due to its low sensitivity and associated costs. However, BAL analysis that includes more than 5% of Langerhans cells staining positive for Cd-1a usually suggests PLCH. As the disease progresses, the utility of BAL decreases due to a decrease in the number of Langerhans cells [4]. Ultimately, a combination of a CT scan, tissue biopsy, and clinical characteristics are used for diagnosis since no definitive criteria have been established for diagnosis.

The most effective treatment for PLCH is smoking cessation, which has shown regression of the disease [14]. Besides smoking cessation, no treatment protocol has been established. Recent studies have shown positive results with the use of cladribine, a chemotherapeutic agent cytotoxic toward monocyte cells [15]. BRAF expression, which is present in nearly half of PLCH patients, is typically responsive to vemurafenib. However, clinical studies show a near-complete relapse after discontinuation of vemurafenib [16]. New research favoring chemotherapeutic drugs require uncommon treatments in comparison to other interstitial diseases, making it imperative these patients are properly diagnosed.

## Case presentation

A 40-year-old African-American male presented to the emergency room for an asthma exacerbation and dyspnea at rest. The patient had been previously admitted a few days prior for another asthma exacerbation and was discharged the next day after stabilization. At this time, the patient had completed a course of trimethoprim-sulfamethoxazole and was taking clindamycin secondary to an actinomycoses infection in the right axilla. Additional medical history included mild cardiomegaly, chronic obstructive pulmonary disease, nephrolithiasis, and obstructive sleep apnea. Family history was positive for asthma and social history, including a 30-year pack history until 2016. The patient worked as a security guard since moving from East Africa in 1997.

On initial examination, his temperature was 101.9 F, blood pressure was 106/57, pulse was 127, respiratory rate 32, and O2 saturation of 92%, which met the Systemic Inflammatory Response Syndrome (SIRS) criteria for sepsis. Subsequent therapy with ipratropium bromide, albuterol, ibuprofen, broad-spectrum antibiotics, and methylprednisolone 60 mg intravenously were given which resulted in stabilization within three hours. Physical examination revealed diffuse expiratory wheezing bilaterally with poor air movement and a right axilla wound with a 5 cm raised incision that was tender to palpation. Bilateral accessory muscle use was noted upon inspiration. There was no distension of the jugular vein, clubbing, or cyanosis. 

Laboratory results immediately after emergency treatment, including arterial blood gas (ABG), white blood cell (WBC), erythrocyte sedimentation rate (ESR), C-reactive protein (CRP), and total IgE can be seen in Table 1. A portable chest x-ray revealed no acute cardiopulmonary disease. This was followed up with a CT without contrast which revealed bilaterally hazy areas of moderately well-defined increased density in both the upper and lower lobes, and predominantly centrally. Chest computed tomography also revealed multiple nonspecific mediastinal nodes, bilateral apical blebs, and prominence of perihilar markings. 

**Table 1:** Pertinent laboratory findings

Test	Result	Reference range
Arterial blood gas (ABG) pH	7.42 pH	7.35 - 7.45 pH
Arterial blood gas (ABG) CO2	33 mmHg	35 - 45 mmHg
Arterial blood gas (ABG) O2	115 mmHg	80 - 100 mmHg
Arterial blood gas (ABG) HCO3	21 mEq/L	22 - 26 mEq/L
White blood cells (WBC)	18,600 x10^9 L	4,400 – 11,000 x 10^9 L
Erythrocyte sedimentation rate (ESR)	46 mm/hr	0 – 15 mm/hr
C-reactive protein (CRP)	6.6 mg/dl	0.1 – 1 mg/dl
Total IgE	8,064 kU/L	<214 kU/L
Sodium	138 mEq/L	135 – 145 mEq/L
Chloride	99 mEq/L	96-106 mEq/L

Although the patient had multiple hospitalizations for asthma exacerbation, this diagnosis was in question due to previous CTs and the current condition, which prompted bronchoalveolar lavage and a biopsy. The patient received a bronchoscopy with video-assisted thoracoscopic surgery, and a chest tube was in place on the left side. BAL wash was negative for the growth of bacteria, fungi, or signs of infection. Pathology findings revealed a focal inflammatory aggregate comprised of acute and chronic inflammation, with scattered staining positive for langerin and CD-1a cells, which raised concern for Langerhans cell histiocytosis. 

**Figure 1:**
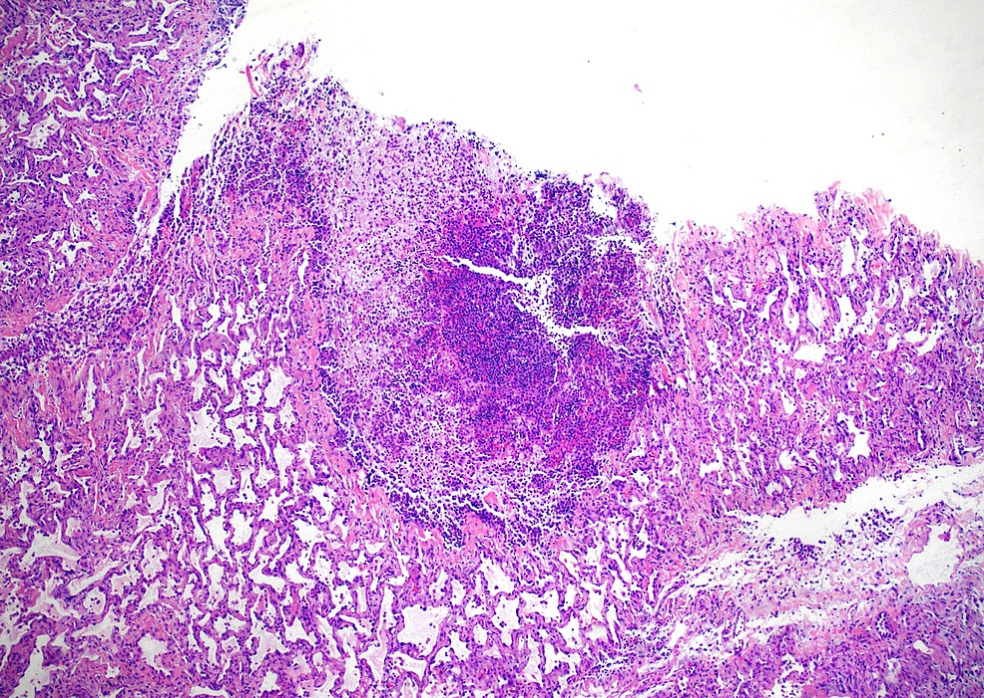
Lung abscess with parenchyma, 4x

**Figure 2:**
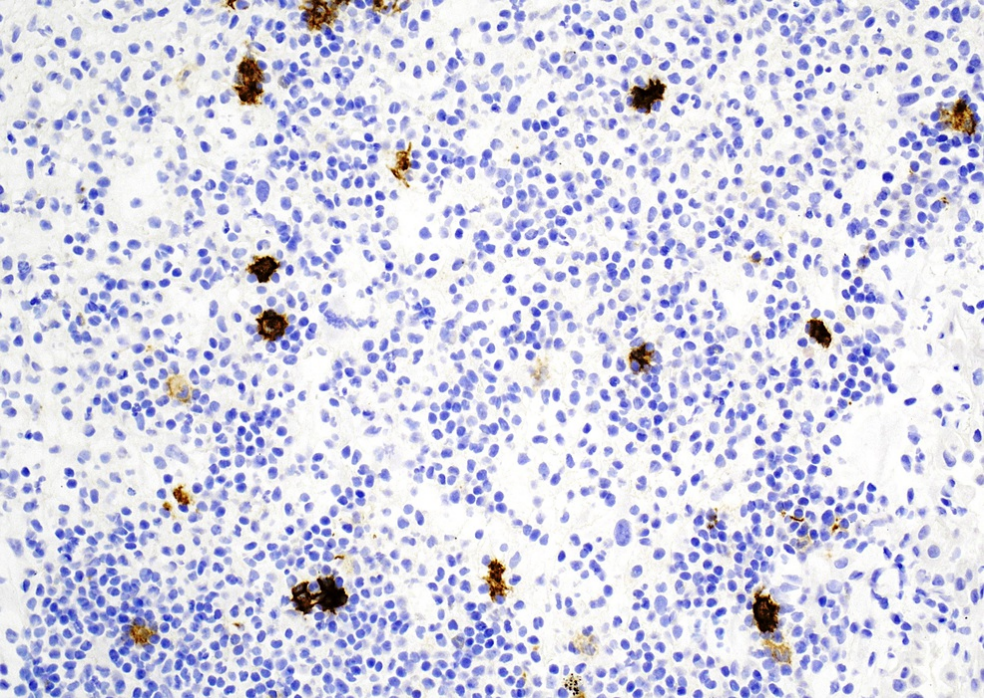
Cd-1a staining positive of Langerhans cells, 20x

**Figure 3:**
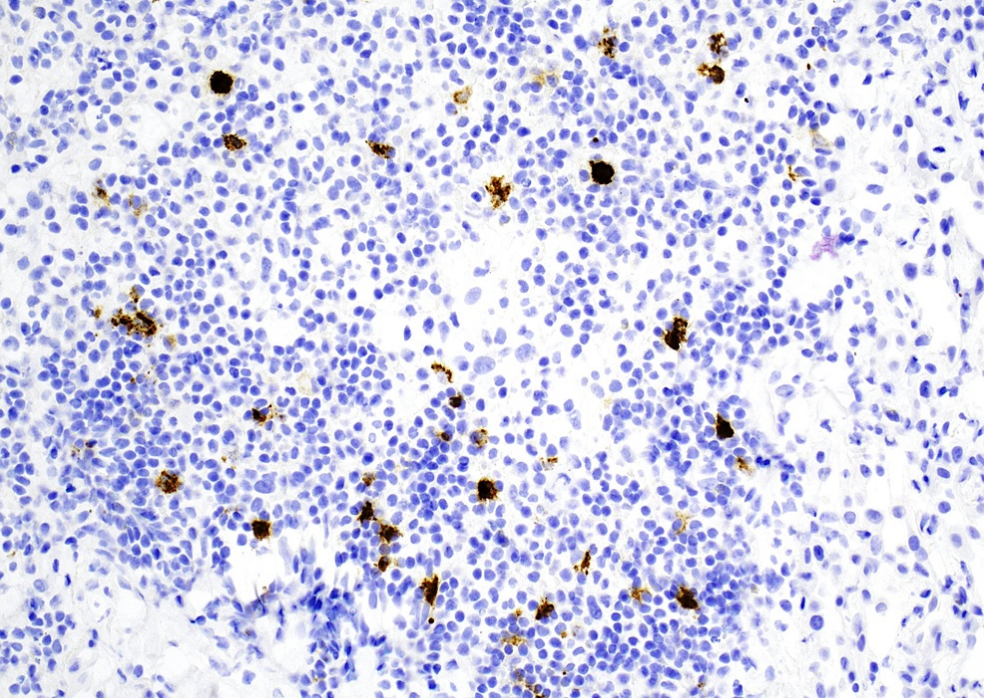
Langerin (CD207) positive staining for Langerhans cells, 20x

The patient was discharged on a tapered prednisone regimen, albuterol, budesonide, salmeterol, morphine for bone pain, and penicillin 500 mg for an actinomycosis skin infection. Pathology slides were forwarded to a tertiary care hospital for further scrutinization of PLCH (Figure 1). After secondary review, the diagnosis was favored to be a focal abscess or empyema due to the lack of definitive clustering of Langerhans cells in the tissue biopsy (Figure 2, Figure 3). Additional follow-up was recommended to definitively rule out PLCH. 

## Discussion

The diagnosis of pulmonary Langerhans cell histiocytosis is commonly made using a combination of CD-1a cells in tissue, bronchoalveolar fluid analysis, and clinical presentation [1,17]. This patient met the criteria for positively stained CD-1a and langerin to detect the presence of Langerhans cells, but had otherwise nonspecific symptoms. However, after follow-up, there was insignificant evidence to definitively rule out PLCH.

Analysis of multiple CT scans and chest x-rays showed no evidence of a lung abscess and worsening of lung parenchyma even after smoking cessation. Additionally, the patient had been on multiple antibiotics, including trimethoprim-sulfamethoxazole, clindamycin, and penicillin, over the last month for an actinomycoses infection. Most abscesses would have been responsive with antibiotic regimens that included clindamycin. This raises concern for further analysis due to the differentiating diagnoses between two hospitals and the discrepancy between pathology and radiography. The lack of evidence on radiography for a lung abscess, in combination with recurrent respiratory flares when taking multiple antibiotics, kept PLCH as a differential.

New diagnosis criteria need to be created for PLCH. Findings of more than 5% of Langerhans cells on BAL have suggested a strong indication of diagnosis, but less than 5% is not a strong enough indication to rule out PLCH [17]. Lung biopsy is generally definitive in the diagnosis of PLCH [18]. However, there is no quantitative diagnosis for PLCH lung tissue biopsies, which poses a problem for situations when trying to identify possible cases of PLCH since BAL is not commonly done. Treatment for PLCH is centered around reducing dyspnea and controlling pulmonary hypertension. New research has shown promising improvement with cladribine in functional class dyspnea and forced expiratory volume in one second [19]. However, cladribine is commonly associated with myelosuppression and typically requires oncologist care. Unclear diagnostic criteria make it difficult to provide optimal treatment by restricting the use of medications without a conclusive diagnosis. 

PLCH is frequently misdiagnosed due to the poor understanding of its clinical characteristics and its similar presentation with other interstitial diseases [5]. A quantitative aspect such as having 5% Langerhans cells on bronchoalveolar lavage could similarly be used on tissue analysis. Sholl et al. showed all cases of PLCH contained greater than 30 langerin (CD207) and Cd-1a cells per high-power field (HPF), with a mean of more than 100 cells per HPF in tissue [20]. Establishing more clear guidelines will allow these patients another category of new medications that might provide them more symptomatic relief than current medical therapies. 

## Conclusions

Patients with PLCH present with non-specific symptoms that can mimic other more common diagnoses such as asthma and chronic obstructive pulmonary disease or may present concurrently with one of these diseases as seen in this patient. Our patient required numerous hospitalizations secondary to presumed asthma exacerbations but remained refractory to the current standard of care and smoking cessation. Eventually, a biopsy allowed for consideration of a secondary diagnosis that may have been compounding the respiratory decline but ultimately was of little utility despite the risk due to contradiction between institutions which did not allow for a conclusive diagnosis. Focusing on quantitative analysis could potentially reduce discrepancies in comparison to current standards which are based on clustering and have prevented undue harm by requiring additional procedures and diagnostic studies. Ultimately, a combination of physical presentation, chest CT, and tissue analysis are all essential to diagnose PLCH. Establishing quantitative criteria for tissue analysis will reduce undue harm from new chemotherapeutic regimens by ensuring treatment for those only truly affected. Further research will allow clinicians to create a standardized approach to minimize the diagnostic dilemma PLCH cases create today.
